# Clinicians Who Practice Primarily in Nursing Homes and the Quality of End-of-Life Care Among Residents

**DOI:** 10.1001/jamanetworkopen.2024.2546

**Published:** 2024-03-15

**Authors:** Arnab K. Ghosh, Mark Aaron Unruh, Hyunkyung Yun, Hye-Young Jung

**Affiliations:** 1 Department of Medicine, Weill Cornell Medical College, Cornell University, New York, New York; 2 Department of Population Health Sciences, Weill Cornell Medical College, Cornell University, New York, New York; 3 Department of Health Services, Policy, and Practice, Brown School of Public Health, Providence, Rhode Island

## Abstract

**Importance:**

Clinician specialization in the care of nursing home (NH) residents or patients in skilled nursing facilities (SNFs) has become increasingly common. It is not known whether clinicians focused on NH care, often referred to as *SNFists* (ie, physicians, nurse practitioners, and physician assistants concentrating their practice in the NH or SNF setting), are associated with a reduced likelihood of burdensome transitions in the last 90 days of life for residents, which are a marker of poor-quality end-of-life (EOL) care.

**Objective:**

To quantify the association between receipt of care from an SNFist and quality of EOL care for NH residents.

**Design, Setting, and Participants:**

This cohort study analyzed Medicare fee-for-service claims for a nationally representative 20% sample of beneficiaries to examine burdensome transitions among NH decedents at the EOL from January 1, 2013, through December 31, 2019. Statistical analyses were conducted from December 2022 to June 2023.

**Exposure:**

Receipt of care from an SNFist, defined as physicians and advanced practitioners who provided 80% or more of their evaluation and management visits in NHs annually.

**Main Outcomes and Measures:**

This study used augmented inverse probability weighting in analyses of Medicare fee-for-service claims for a nationally representative 20% sample of beneficiaries. Main outcomes included 4 measures of burdensome transitions: (1) hospital transfer in the last 3 days of life; (2) lack of continuity in NHs after hospitalization in the last 90 days of life; (3) multiple hospitalizations in the last 90 days of life for any reason or any hospitalization for pneumonia, urinary tract infection, dehydration, or sepsis; and (4) any hospitalization in the last 90 days of life for an ambulatory care–sensitive condition.

**Results:**

Of the 2 091 954 NH decedents studied (mean [SD] age, 85.4 [8.5] years; 1 470 724 women [70.3%]), 953 722 (45.6%) received care from SNFists and 1 138 232 (54.4%) received care from non-SNFists; 422 575 of all decedents (20.2%) experienced a burdensome transition at the EOL. Receipt of care by an SNFist was associated with a reduced risk of (1) hospital transfer in the last 3 days of life (−1.6% [95% CI, −2.5% to −0.8%]), (2) lack of continuity in NHs after hospitalization (−4.8% [95% CI, −6.7% to −3.0%]), and (3) decedents experiencing multiple hospitalizations for any reason or any hospitalization for pneumonia, urinary tract infection, dehydration, or sepsis (−5.8% [95% CI, −10.1% to −1.7%]). There was not a statistically significant association with the risk of hospitalization for an ambulatory care–sensitive condition in the last 90 days of life (0.0% [95% CI, −14.7% to 131.7%]).

**Conclusions and Relevance:**

This study suggests that SNFists may be an important resource to improve the quality of EOL care for NH residents.

## Key Points

**Question:**

Do clinicians who practice primarily in skilled nursing facilities (SNFs) or nursing homes (*SNFists* [ie, physicians, nurse practitioners, and physician assistants concentrating their practice in the SNF or nursing home setting]) provide higher-quality end-of-life care compared with other clinicians?

**Findings:**

This cohort study comprised 2 091 954 nursing home decedents, including 953 722 residents who received care from SNFists and 1 138 323 decedents who received care from non-SNFists. Residents who received care from SNFists had a reduced risk of experiencing burdensome transitions at the end of life, such as hospital transfer in the last 3 days of life or lack of continuity in nursing homes after hospitalization.

**Meaning:**

This study suggests that SNFists may be an important resource to improve the quality of end-of-life care for nursing home residents.

## Teaser

This cohort study of more than 2 million nursing home residents examines the association between receipt of care from a physicians, nurse practitioners, or physician assistants concentrating their practice in the nursing home or skilled nursing facility SNF setting (ie, an SNFist) and quality of end-of-life care for nursing home residents.

## Introduction

Nursing home (NH) residents at the end of life (EOL) frequently experience burdensome transitions,^1^ reflecting poor quality of care. Burdensome transitions are disruptive to residents^2^ and lower quality of life through increased risk of feeding tube use,^3^ intensive care unit stays,^4^ late enrollment in hospice,^5^ and receipt of care that is not concordant with the resident’s wishes.^6^

During the past 2 decades, the proportion of clinicians (ie, physicians, nurse practitioners, and physician assistants) who practice primarily in NHs or skilled nursing facilities (SNFs), frequently termed *SNFists* (ie, physicians, nurse practitioners, and physician assistants concentrating their practice in the NH or SNF]setting), has increased at a rapid pace.^7,8^ Most NHs in the US now use SNFists.^9^ However, it is unclear whether the use of SNFists improves the quality of EOL care for NH residents. Given the clinical complexity and frailty of NH residents, SNFists’ focus on the NH setting may provide them with stronger clinical, organizational, and regulatory knowledge for these patients and thus lower the risk of burdensome transitions at the EOL.^10,11^ Alternatively, such specialization may increase fragmentation in care delivered to NH residents, leading to increased risk of EOL transfers.^12^

Using a nationally representative sample of Medicare claims, we examined the association between receipt of care by SNFists and burdensome transitions of care in the last 90 days of life for NH residents. We hypothesized that NH residents who received care from SNFists had a lower risk of burdensome transitions at the EOL compared with residents who received care from other clinicians.

## Methods

### Study Population, Cohort Definition, and Data Sources

The cohort study population included decedents aged 65 years or older who were long-stay NH residents, defined as Medicare beneficiaries enrolled in Medicare Parts A and B with stays in the same facility for at least 100 days, with no more than 10 consecutive days outside the NH, prior to death.^13^ This study was approved by the Weill Cornell Medicine institutional review board. The Centers for Medicare & Medicaid Services Privacy Board approved a waiver of informed consent because research using administrative datasets cannot practicably be carried out without such a waiver. This article followed the Strengthening the Reporting of Observational Studies in Epidemiology (STROBE) reporting guideline for observational studies.

We used Medicare fee-for-services claims for a 20% nationally representative sample of beneficiaries for January 1, 2013, through December 31, 2019. These included Medicare claims for inpatient and outpatient services, as well as the Master Beneficiary Summary File with the Chronic and Disabling Conditions Segments. These data were linked to Minimum Data Set assessments, NH-level data from the Long-Term Care: Facts on Care in the US (LTCFocus) database and Medicare’s Care Compare for NHs, and clinician-level data from the Medicare Accountable Care Organization (ACO) Provider file. In addition, county-level market information was merged from the Area Health Resources File.

The clinical characteristics of the NH residents, including activities of daily living score and Cognitive Function Scale, along with their marital status were derived from the Minimum Data Set.^14^ Minimum Data Set assessments are federally mandated for all NH residents and include information on demographic characteristics, diagnoses, and measures of both physical and cognitive functional status. Resident characteristics; enrollment status for Medicare Parts A and B, including dual eligibility status for Medicare and Medicaid and reason for Medicare entitlement; and chronic conditions were obtained from the Master Beneficiary Summary File. These data were merged with NH characteristics from LTCFocus and Care Compare using facility identifers.^15^ Clinicians’ ACO participation was derived from the ACO Provider File and aggregated to the NH level as a percentage of clinicians who were ACO participants. Nursing home Five Star ratings were derived from Care Compare. County-level clinician labor market characteristics were obtained from the Area Health Resources File (eFigure in Supplement 1).

### Outcomes: Burdensome Transitions

Informed by the previous literature,^1^ burdensome transitions in the last 90 days of life were defined as follows: (1) hospital transfer in the last 3 days of life; (2) lack of continuity in NHs after hospitalization (eg, transfer from one NH to the hospital and then to a different NH); (3) multiple hospitalizations for any reason or any hospitalization for pneumonia, urinary tract infection, dehydration, or sepsis (defined using *International Classification of Diseases, Ninth Revision* or *International Statistical Classification of Diseases and Related Health Problems, Tenth Revision* codes (eTable in Supplement 1); or (4) any hospitalization for an ambulatory care–sensitive condition as defined by the Agency for Healthcare Research and Quality.^16^ Indicators for each of these 4 types of transitions were used as outcomes.

### Exposure: Receipt of Care From an SNFist

We defined SNFists as physicians and advanced practitioners (nurse practitioners and physician assistants) who provided 80% or more of their evaluation and management visits in the NH setting.^7,8,17^ Physicians in the sample were limited to generalists (family practice, general practice, geriatric medicine, internal medicine, and physical medicine and rehabilitation). In addition, we required clinicians to have at least 100 service lines in claims annually and to have made 10 or more visits in NHs in a given year. Residents were attributed annually to the clinician with a plurality of their evaluation and management visits.^18^

### Other Independent Variables

Resident demographic characteristics included age, sex, race and ethnicity, marital status, reason for Medicare entitlement, and dual eligibility for Medicare and Medicaid. Our analysis determined race and ethnicity using the race and ethnicity variable from the Medicare Beneficiary Summary File. We assessed race and ethnicity because of well-known racial and ethnic disparities in EOL care.^19^ Residents’ clinical conditions included indicators for common chronic conditions, activities of daily living score (0- to 28-point physical function score, where higher scores indicate decreased independence), and Cognitive Function Scale (ranging from 1 to 4, where higher scores indicate worse cognitive impairment). Facility-level covariates included the total number of beds, proportion of White residents, indicators of for-profit status, being part of a multifacility chain, being associated with a hospital, presence of Alzheimer disease care unit, proportions of Medicare- and Medicaid-covered residents, percentage of clinicians who were ACO members, rural location, and overall Care Compare Five Star rating. The number of primary care clinicians per 100 000 population was used as a physician labor market characteristic.

### Statistical Analysis

Statistical analyses were conducted from December 2022 to June 2023. Unadjusted comparisons based on whether a resident received care from an SNFist or a non-SNFist were made using the 2-sample *t* test for continuous variables and the Pearson χ^2^ test for categorical variables.

We performed a decedent-level, complete-case analysis to estimate the association between receipt of care from an SNFist compared with a non-SNFist and risk of burdensome transitions at the EOL. We used a hierarchical multivariable logistic regression model with augmented inverse probability weighting (AIPW), a causal inference technique that uses the Rubin causal effects framework to examine the probability of an outcome of interest when the counterfactual outcome is not always observed.^20^ Augmented inverse probability weighting combines the properties of both a regression-based estimator of the outcome (ie, the probability of a burdensome transition) and the inverse probability–weighted (IPW) estimator that determines the probability of a resident receiving care from an SNFist. The AIPW is a doubly robust estimator, meaning that if either estimator is misspecified, the mean treatment effect converges to the true effect as the number of observations increases.^20^

Both the regression model and the IPW model included the resident, NH, and market characteristics. To account for variation of practices within NHs and also for state-based variation in SNFist practice patterns over time,^8^ individual intercepts were used in the regression models by including indicators for each NH and the state year. The AIPW model estimated the mean treatment effect, denoting the percentage difference of the average NH resident who received care from an SNFist experiencing a burdensome transition in the last 90 days of life compared with an equivalent NH resident who received care from a clinician who was not an SNFist.

For each model, balance of the covariates was assessed using the covariate balancing propensity score test derived by Imai and Ratkovic.^21^ All *P* values were from 2-sided tests and, for each model, we considered results with *P* < .05 to be statistically significant. All analyses were conducted using Stata, version 17 (StataCorp LLC).

## Results

### Unadjusted Comparisons

Table 1 presents descriptive statistics for decedents, NHs, and markets. The study population included 2 091 954 decedents (mean [SD] age, 85.4 [8.5] years; 1 470 724 women [70.3%]). Compared with those who received care from non-SNFists, NH residents who received care from SNFists were somewhat younger (proportion aged ≥85 years: 543 711 [57.0%] vs 686 527 [60.3%]; *P* < .001), less likely to be female (662 682 [69.5%] vs 808 042 [71.0%]; *P* < .001), had higher rates of end-stage kidney disease (22 378 [2.4%] vs 21 963 [1.9%]; *P* < .001), were more likely to be dually eligible for Medicare and Medicaid (770 217 [80.8%] vs 879 802 [77.3%]; *P* < .001), and had higher rates of common chronic conditions, including Alzheimer disease and related dementias (811 615 [85.1%] vs 940 841 [82.7%]; *P* < .001). Nursing home residents treated by SNFists were more likely to be in larger NHs (mean [SD] number of beds, 144.3 [84.7] vs 128.8 [74.5]; *P* < .001), for-profit facilities (mean [SD], 70.8% [47.3%] vs 65.9% [45.4%]; *P* < .001), and those with higher mean (SD) proportions of residents covered by Medicare (mean [SD], 14.7% [10.1%] vs 14.2% [10.0%]; *P* < .001) and Medicaid (mean [SD], 62.0% [18.7%] vs 60.3% [19.9%]; *P* < .001) and with lower percentages of clinicians participating in an ACO (181 888 [19.1%] vs 308 305 [27.1%]; *P* < .001).

**Table 1.** Characteristics of Nursing Home Decedents Who Received Care by SNFists vs Non-SNFists^a^

Characteristic	Non-SNFist, No. (%) (n = 1 138 232)	SNFist, No. (%) (n = 953 722)	*P* value^b^
Resident demographics			
Age group			
65-69 y	52 188 (4.6)	52 097 (5.5)	<.001
70-74 y	84 840 (7.5)	82 136 (8.6)	<.001
75-79 y	124 418 (10.9)	111 946 (11.7)	<.001
80-84 y	190 259 (16.7)	163 772 (17.2)	<.001
≥85 y	686 527 (60.3)	543 771 (57.0)	<.001
Sex			
Female	808 042 (71.0)	662 682 (69.5)	<.001
Male	330 190 (29.0)	291 040 (30.5)	<.001
Married	243 000 (21.7)	205 405 (22.0)	<.001
Rurality			
Metropolitan	823 070 (72.1)	839 655 (88.0)	<.001
Nonmetropolitan	220 222 (19.4)	85 902 (9.0)	
Rural	94 940 (8.3)	28 165 (3.0)	
Racial and ethnic group			
Asian	13 737 (1.2)	9158 (1.0)	<.001
Black	98 028 (8.6)	103 750 (10.9)	
Hispanic	16 821 (1.5)	12 640 (1.3)	
White, non-Hispanic	994 864 (87.4)	816 644 (85.6)	
Other^c^	14 782 (1.3)	11 530 (1.2)	
Reason for Medicare entitlement			
Age	951 659 (83.6)	782 113 (82.0)	<.001
Disability	183 436 (16.1)	168 434 (17.7)	
End-stage kidney disease	21 963 (1.9)	22 378 (2.4)	
Dually eligible for Medicare-Medicaid	879 802 (77.3)	770 217 (80.8)	<.001
Decedent clinical characteristics			
Common chronic conditions			
Alzheimer disease and related dementias	940 841 (82.7)	811 615 (85.1)	<.001
Chronic heart failure	536 608 (47.1)	466 090 (48.9)	<.001
Chronic kidney disease	498 633 (43.8)	459 756 (48.2)	<.001
Chronic obstructive pulmonary disease	304 722 (26.8)	263 917 (27.7)	<.001
Diabetes	478 237 (42.0)	419 864 (44.0)	<.001
Coronary heart disease	582 991 (51.2)	502 529 (52.7)	<.001
Stroke or transient ischemic attack	178 242 (15.7)	154 649 (16.2)	<.001
Hepatic failure	45 450 (4.0)	44 166 (4.6)	<.001
Obesity	131 176 (11.5)	121 101 (12.7)	<.001
Schizophrenia	60 225 (5.3)	59 002 (6.2)	<.001
Activities of daily living score, mean (SD)	15.8 (6.6)	16.1 (6.4)	<.001
Cognitive Function Scale, mean (SD)	2.09 (0.8)	2.09 (0.8)	>.99
Facility or clinician characteristics			
No. of beds, mean (SD)	128.8 (74.5)	144.3 (84.7)	<.001
White residents, mean (SD), %	83.3 (0.02)	81.1 (0.02)	<.001
For-profit status, mean (SD), %	65.9 (0.0004)	70.8 (0.0005)	<.001
Associated with hospital, mean (SD), %	3.3 (0.0002)	1.8 (0.0001)	<.001
Multifacility nursing home, mean (SD), %	52.1 (0.0004)	57.6 (0.0005)	<.001
Alzheimer unit present, mean (SD), %	20.8 (0.0003)	22.2 (0.0004)	<.001
Clinicians affiliated with an Accountable Care Organization	308 305 (27.1)	181 888 (19.1)	<.001
Medicare-covered residents, mean (SD), %	14.2 (0.009)	14.7 (0.01)	<.001
Medicaid-covered residents, mean (SD), %	60.3 (0.02)	62.0 (0.02)	<.001
Medicare Five-Star Facility rating, No. (%)			
1 (Lowest)	8840 (13.0)	9792 (14.4)	<.001
2	13 260 (19.5)	13 601 (20.7)	
3	12 785 (18.8)	12 649 (18.6)	
4	16 864 (24.8)	16 456 (24.2)	
5 (Best)	16 252 (23.9)	15 096 (22.2)	
Physician labor market characteristics			
No. of primary care professionals per 100 000 population, mean (SD)	582.6 (1229.7)	652.9 (980.6)	<.001
Outcomes			
Burdensome transitions			
Hospital transfer in last 3 d of life	178 419 (15.7)	145 474 (15.3)	<.001
Lack of continuity in nursing home after hospitalization in last 90 d of life	45 091 (4.0)	35 732 (3.8)	<.001
Multiple hospitalizations for any reason or any hospitalization for pneumonia, urinary tract infection, dehydration, or sepsis in last 90 d of life	9351 (0.8)	6689 (0.7)	<.001
Admissions for ambulatory care–sensitive condition in last 90 d of life	740 (0.07)	414 (0.04)	<.001

Abbreviation: SNFist, physicians, nurse practitioners, and physician assistants concentrating their practice in the nursing home or skilled nursing facility setting.

^a^ SNFist defined as physicians and advanced practitioners who provided 80% or more of their billing codes for evaluation and management in the nursing home setting.

^b^ Comparisons were made using the 2-sample *t* test for continuous variables and the Pearson χ^2^ test for categorical variables.

^c^ All beneficiaries defined in the Master Beneficiary Summary File as being Asian, North American Native, other, or unknown.

Overall, 422 575 of all decedents (20.2%) in our sample experienced at least 1 type of burdensome transition at the EOL. Those who received SNFist care had, on average, a lower unadjusted rate of each type of burdensome transition compared with those who received care from non-SNFists (hospital transfer in last 3 days of life: 145 474 [15.3%] vs 178 419 [15.7%]; lack of continuity in NHs after hospitalization: 35 732 [3.8%] vs 45 091 [4.0%]; multiple hospitalizations for any reason or any hospitalization for pneumonia, urinary tract infection, dehydration, or sepsis: 6689 [0.7%] vs 9351 [0.8%]; and hospitalization for an ambulatory care–sensitive condition: 414 [0.04%] vs 740 [0.07%]; all *P* < .001) (Table 1).

### Adjusted Comparisons

Adjusted estimates are reported in Table 2. Compared with NH residents who received care from non-SNFist clinicians, residents who received care from an SNFist were 1.6% less likely to experience any hospital transfer in the last 3 days of life (95% CI, −2.5% to −0.8%; *P* < .001). In the last 90 days of life, residents who received care from SNFists were 4.8% (95% CI, −6.7% to −3.0%) less likely to experience a lack of continuity in NHs and 5.8% (95% CI, −10.1% to −1.7%) less likely to have multiple hospitalizations or at least 1 hospitalization for pneumonia, urinary tract infections, dehydration, or sepsis. There was not a statistically significant difference (0.0% [95% CI, −14.7% to 131.7%]) in the risk of a hospital admission for an ambulatory care–sensitive condition in the last 90 days of life.

**Table 2.** Adjusted Differences in Rates of Burdensome Transitions at the End of Life Among NH Decedents Who Were Cared for by SNFists vs Non-SNFists^a^^,^^b^

Adjusted difference	Type of burdensome transition			
	Hospital transfer in last 3 d of life (95% CI)	Lack of continuity in NH after hospitalization in last 90 d of life (95% CI)	Multiple hospitalizations or any hospitalization for pneumonia, UTI, dehydration, or sepsis (95% CI)	Hospital admission for an ambulatory care–sensitive condition in the last 90 d of life (95% CI)
Proportion of NH decedents, SNFist vs non-SNFist care	−0.3 (−0.4 to −0.1)	−0.2 (−0.3 to −0.1)	−0.05 (−0.08 to −0.01)	0.00 (−0.01 to 0.1)
Mean adjusted proportion of NH decedents, non-SNFist care	15.8 (15.8 to 15.9)	4.0 (4.0 to 4.1)	0.8 (0.8 to 0.8)	0.07 (0.06 to 0.07)
Likelihood, SNFist vs non-SNFist care	−1.6 (−2.5 to −0.8)	−4.8 (−6.7 to −3.0)	−5.8 (−10.1 to −1.7)	0.0 (−14.7 to 131.7)

Abbreviations: NH, nursing home; SNFist, physicians, nurse practitioners, and physician assistants concentrating their practice in the nursing home or skilled nursing facility setting; UTI, urinary tract infection.

^a^ Inverse probability weighted estimates adjusted for resident, NH, and market characteristics. Regression models also include indicators for each NH and the state year.

^b^ SNFist defined as physicians and advanced practitioners who provided 80% or more of their billing codes for evaluation and management in the NH setting.

## Discussion

In this national cohort study of NH decedents, we found that receipt of care from SNFists was associated with a reduced likelihood of experiencing 3 of 4 types of burdensome transitions, suggesting that these clinicians may play an important role in improving the EOL care for this population. To our knowledge, this is the first study to examine the association between care provided by SNFists and the quality of EOL care.

Previous studies examining NH residents receiving postacute and long-term care have described associations of receipt of SNFist care with reduced rehospitalization rates,^18^ long-term use of antipsychotics, and indwelling bladder catheter use.^22^ Our findings extend the literature by suggesting that SNFists’ strengths may facilitate better EOL care.^12,23^ These strengths include familiarity with the common clinical conditions faced by NH residents,^22^ the unique regulatory environment of NHs, and the likelihood of better communication between residents, families, and NH staff.^12,23^ Moreover, our findings are of a similar magnitude and direction as those from a post hoc analysis, the Pragmatic Trial of Video Education in Nursing Homes (PROVEN) published by Moyo et al,^24^ that demonstrated a 1.7% (95% CI, −3.2% to −0.1%) reduction in burdensome transitions in the last 90 days of life with the use of video-assisted advance care planning. However, our results are lower in magnitude than those reported by Miller et al.^25^ In their propensity score–matched retrospective cohort study that examined the role of palliative care consultations in the last 6 months of life, there was an estimated reduction in burdensome transitions at the EOL (16.2% [95% CI, 12.7%-18.6%]) compared with matched controls (28.2% [95% CI, 25.8%-30.6%]).

Although further research is required to better characterize the mechanisms and the degree to which SNFists provide holistic care across various domains, including multimorbidity, functional and cognitive impairment, and frailty of NH residents, there are several policy implications that can be drawn from these findings. First, as the proportion of clinicians providing care in NHs who are SNFists continues to increase^8^ and active recruitment of them by NHs nationwide continues,^9^ our findings underscore the growing importance of SNFists in the delivery of EOL care to frail US individuals with advanced age and associated comorbidities, particularly those with dementia, as two-thirds of all deaths related to Alzheimer disease occur in NHs.^26^

Second, the increase in SNFists reflects not only a growing need but a pattern of site-specific specialization similar to the practice of hospital medicine, and it is being increasingly recognized as such.^11^ Several institutions, including the American Medical Directors Association–The Society for Post-Acute and Long-Term Care Medicine, provide structured training through certification in recognition of the unique skills required to expertly care for NH residents. However, most of the growth among clinicians focusing on NH care stems from advanced practitioners, which may reflect an unmet need in the supply of physician SNFists.^8^ This differential growth among clinicians may reflect a lack of exposure to the NH environment during medical training, poorer financial incentives to practice in this setting, and a lack of a clear pathway to specializing in NH care. Medical schools and residency training programs led by the Accreditation Council for Graduate Medical Education may consider ways of providing these experiences for all primary care adult specialties to potentially increase the number of physicians focusing on NH care. Furthermore, such training, and specifically EOL care training, should be provided to other health care professionals, particularly nurse practitioners and physician assistants, who practice in the NH setting.

Third, historically, the quality of EOL care in NHs has focused on the use of in-hospital care and advance care planning. However, our findings indicate that 1 in 5 NH decedents experiences a burdensome transition at the EOL that, compared with earlier estimates, indicates little to no reduction in the rates of these events during the previous decade or more.^1^ This finding suggests that greater emphasis on reducing their frequency is needed by NHs, payers, and policymakers.

### Limitations

Our results should be considered in light of the following limitations. First, although we used a rigorous AIPW approach for estimation of the association between receipt of care from an SNFist and the likelihood of a burdensome transition at the EOL, we cannot rule out the possibility of unobserved confounding. For example, decisions about EOL care may be informed by teams of health professionals and not just the SNFists, whose knowledge and experience may vary across NHs. Therefore, estimates should be viewed as associations and not causal effects. Second, we did not have information on the presence of advance directives or resident preferences for care at the EOL. Third, our estimates indicate modestly lower rates of burdensome transitions at the EOL associated with care provided by SNFists vs non-SNFists. However, the estimated differences translate to approximately 120 000 avoided hospitalizations in the last 90 days of life among the 2 million decedents in our sample.

## Conclusions

This cohort study suggests that burdensome transitions at the EOL among NH residents are frequent and may be reduced through care provided by SNFists. These findings highlight the potential of SNFists to improve the quality of EOL care for this vulnerable and medically complex population.

## Notes

Supplement 1. **eTable.** List of *International Classification of Diseases Versions 9* and *10* Codes for Pneumonia, Urinary Tract Infection, Dehydration, and Sepsis **eFigure.** Exclusion Cascade

Supplement 2.
Data Sharing Statement
