# Supplementary material for: Clinicians Who Practice Primarily in Nursing Homes and the Quality of End-of-Life Care Among Residents
Source: JAMA Netw Open. 2024 Mar 15;7(3):e242546. doi: 10.1001/jamanetworkopen.2024.2546 (PMC10943410; doi:10.1001/jamanetworkopen.2024.2546)
Supplement: Supplement 1. — eTable. List of International Classification of Diseases Versions 9 and 10 Codes for Pneumonia, Urinary Tract Infection, Dehydration, and Sepsis eFigure. Exclusion Cascade [file jamanetwopen-e242546-s001.pdf]

Supplementary Online Content

Ghosh AK, Unruh MA, Yun H, Jung HY. Clinicians who practice primarily in nursing homes and the quality of end-of-life care among residents. *JAMA Netw Open.* 2024;7(3):e242546. doi:10.1001/jamanetworkopen.2024.2546

eTable. List of *International Classification of Diseases Versions 9* and *10* Codes for Pneumonia, Urinary Tract Infection, Dehydration, and Sepsis

eFigure. Exclusion Cascade

This supplementary material has been provided by the authors to give readers additional information about their work.

© 2024 Ghosh AK et al. *JAMA Network Open.*

eTable. List of *International Classification of Diseases Versions 9* and *10* Codes for Pneumonia, Urinary Tract Infection, Dehydration, and Sepsis

	**ICD-9**	**ICD-10**
**Pneumonia**	00322 0203 0204 0205 0212 0221 0310 0391 0521 0551 0730 0830 1124 1140 1144 1145 11505 11515 11595 1304 1363 4800 4801 4802 4803 4808 4809 481 4820 4821 4822 4823 48230 48231 48232 48239 4824 48240 48241 48242 48249 4828 48281 48282 48283 48284 48289 4829 483 4830 4831 4838 4841 4843 4845 4846 4847 4848 485 486 5130 5171	B330 J09X1 J09X2 J1000 J1001 J1008 J101 J1100 J1108 J120 J121 J122 J123 J1281 J1289 J129 J13 J14 J153 J154 J157 J159 J160 J168 J180 J181 J188 J189 J920 J929 J941 J949 R091
**Urinary Tract Infection**	03284 59000 59001 59010 59011 5902 5903 59080 59081 5909 5950 5951 5952 5953 5954 59581 59582 59589 5959 5970 59780 59781 59789 59800 59801 5990	A1810 A1811 A1812 A1813 A3685 A5275 A5401 A5611 A5619 A985 B650 B901 N10 N110 N118 N119 N12 N135 N136 N151 N2884 N2885 N2886 N3000 N3001 N3010 N3011 N3020 N3021 N3030 N3031 N3080 N3081 N3090 N3091 N340 N342 N343 N390
**Dehydration**	275.6	E86
**Sepsis**	7907 038.XX 995.9X 785.52	A021, A227, A267, A327, A400, A401, A403, A408, A409, A4101, A4102, A411, A412, A413, A414, A4150, A4151, A4152, A4153, A4159, A4181, A4189, A419, A427, A5486, B377, R6520, R6521.

© 2024 Ghosh AK et al. *JAMA Network Open.*

eFigure. Exclusion Cascade

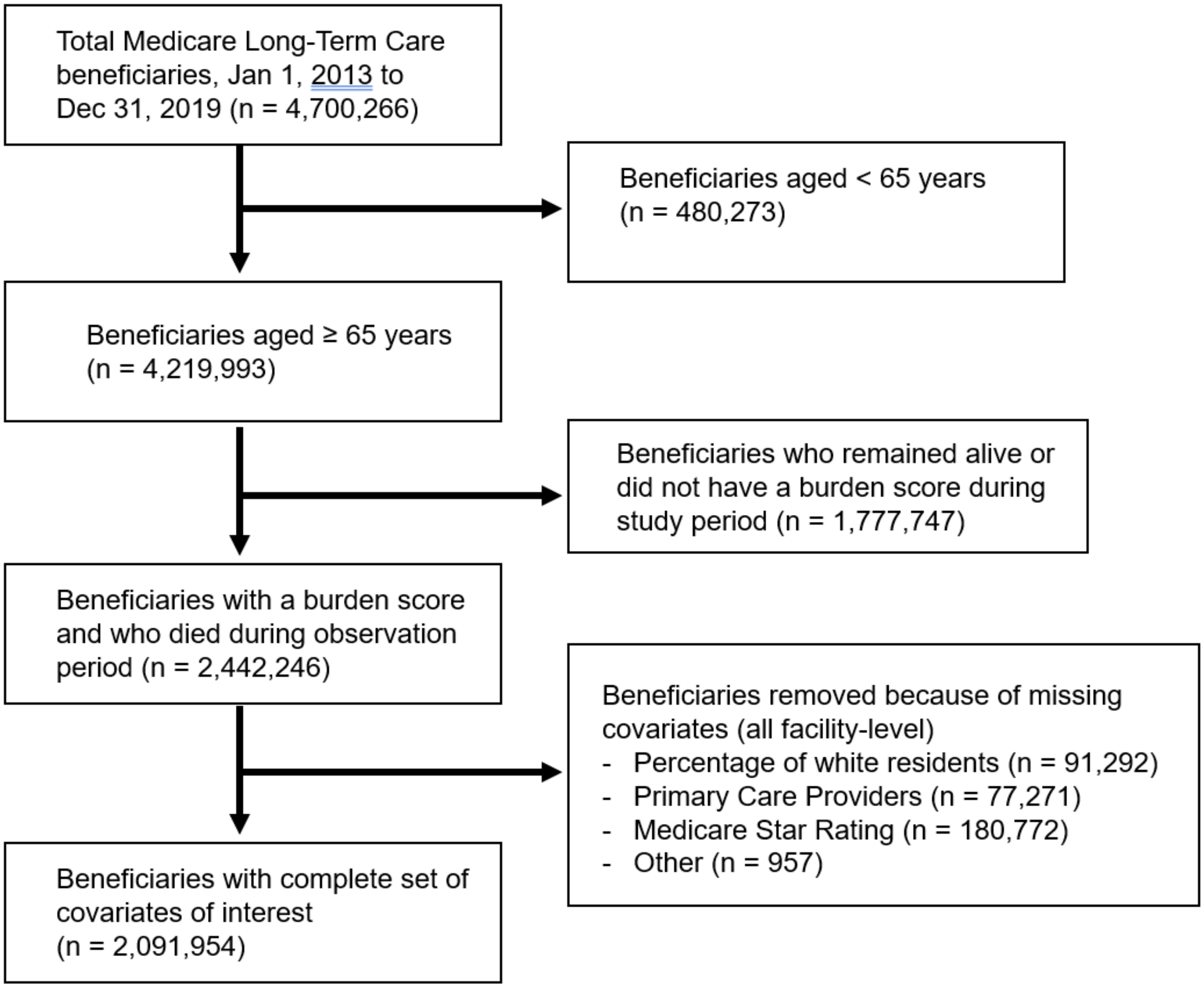

© 2024 Ghosh AK et al. *JAMA Network Open.*
