# Supplementary material for: Clinicians Who Practice Primarily in Nursing Homes and the Quality of End-of-Life Care Among Residents
Source: JAMA Netw Open. 2024 Mar 15;7(3):e242546. doi: 10.1001/jamanetworkopen.2024.2546 (PMC10943410; doi:10.1001/jamanetworkopen.2024.2546)
Supplement: Supplement 2. — Data Sharing Statement [file jamanetwopen-e242546-s002.pdf]

Data Sharing Statement

Ghosh. Clinicians Who Practice Primarily in Nursing Homes and the Quality of End-of-Life Care Among Residents. *JAMA Netw Open*. Published March 15, 2024. doi:10.1001/jamanetworkopen.2024.2546

Data
Data available: No

Additional Information
Explanation for why data not available: This is Medicare data governed by a DUA
